# Neuronal avalanches change from wakefulness to deep sleep - a study of intracranial depth recordings in humans

**DOI:** 10.1186/1471-2202-14-S1-P237

**Published:** 2013-07-08

**Authors:** Viola Priesemann, Mario Valderrama, Michael Wibral, Michel Le Van Quyen

**Affiliations:** 1 Max Planck Institute for Brain Research, Frankfurt, 60486, Germany; 2 Frankfurt Institute for Advanced Studies, Frankfurt, 60438, Germany; 3 University of Los Andes, Bogotá, Colombia; 4 Goethe University, MEG Unit, Frankfurt, 60325, Germany; 5 Hôpital de la Pitié-Salpêtrière, Paris, 75013, France

## 

Neuronal dynamics differs between wakefulness and sleep stages, so does the cognitive state [1]. In contrast, a single attractor state, called self-organized critical (SOC), has been proposed to govern human brain dynamics for its optimal information coding and processing capabilities [2]. Here we address two open questions: First, does the human brain always operate in this computationally optimal state, even during deep sleep? Second, previous evidence for SOC was based on activity *within *single brain areas [3-5], however, the interaction *between *brain areas may be organized differently. Here we asked whether the interaction *between *brain areas is SOC.

We addressed these questions by characterizing neuronal avalanches [3] - spatiotemporal waves of enhanced activity - from up to 61 local field potential (LFP) channels of intracranial depth recordings (5 human patients, two recording nights each, summing up to ~100h of recordings). The recording contacts were distributed inside the entire brain. Note that ~60 contacts are sufficient to avoid major subsampling effects: Subsampling may heavily distort results in SOC systems due to an insufficient number of sampling sites [5]. In addition, we compared the experimental results to results from a subsampled SOC model of integrate- and fire neurons, which can be tuned to the sub- and supercritical regime.

We show that avalanche distributions closely follow a power law - the hall mark feature of SOC systems. This result held for each vigilance state, and independent of the threshold and the temporal scale. This indicates first that the interaction *between *brain areas are close to SOC, and second that the dynamics of all cognitive states, from wakefulness to deep sleep are close to SOC. Minor differences between cognitive states are, however, reflected in the avalanche distributions: Slow wave sleep (s3/s4) showed larger and longer neuronal avalanches than REM sleep, while wakefulness showed intermediate ones (p < 0.05). The SOC neuronal model, together with the data, suggested first that these differences are mediated by global but tiny changes in synaptic strength, and second, that the changes with vigilance states reflect small deviations from criticality to the subcritical regime, implying that the human brain does not operate in the SOC state proper - contrary to previous believes. Independent of criticality, the analysis confirmed that slow wave sleep shows increased correlations between cortical areas, and revealed that REM sleep shows more fragmented cortical dynamics.
